# Supplementary material for: Neurocognitive Inhibitory Control Ability Performance and Correlations with Biochemical Markers in Obese Women
Source: Int J Environ Res Public Health. 2020 Apr 15;17(8):2726. doi: 10.3390/ijerph17082726 (PMC7216261; doi:10.3390/ijerph17082726)
Supplement: Supplementary file 1 [file ijerph-17-02726-s001.pdf]

Supplementary materials

Table. Correct trials and count trials for ERP analysis in Go/Nogo and Stroop tasks.

	Task trials	Correct trials count for RT	Correct trials count for RT (%)	Trials count for ERP analysis	Trials count for ERP analysis (%)
**Go/Nogo**					
Go OG	160	159.81 ± 0.40	99.88 ± 0.25	149.19 ± 9.02	93.36 ± 5.66
Go CG	160	159.85 ± 0.46	99.90 ± 0.29	150.92 ± 11.20	94.42 ± 6.98
Nogo OG	40	39.38 ± 1.01	98.46 ± 2.65	37.65 ± 2.65	95.62 ± 6.43
Nogo CG	40	39.31 ± 1.01	98.27 ± 2.53	37.35 ± 2.24	95.05 ± 5.86
**Stroop**					
Congruent OG	100	99.15 ± 0.93	99.15 ± 0.92	93.15 ± 11.71	93.98 ± 11.94
Congruent CG	100	98.88 ± 1.66	98.88 ± 1.66	92.69 ± 16.52	93.66 ± 16.47
Incongruent OG	100	97.00 ± 2.65	97.00 ± 2.65	90.23 ± 11.37	93.04 ± 11.40
Incongruent CG	100	97.54 ± 3.08	97.54 ± 3.08	89.92 ± 15.33	92.16 ± 15.43

Values are means ± SD. OG, Obese group; CG, Control group.
Correct trials count for RT (%) = 100 * Correct trials count for RT/ Task trials;
Count trials for ERP analysis (%) = 100 * Count trials for ERP analysis / Correct trials count.
